# Prediction of the Spinal Musculoskeletal Loadings during Level Walking and Stair Climbing after Two Types of Simulated Interventions in Patients with Lumbar Disc Herniation

**DOI:** 10.1155/2019/6406813

**Published:** 2019-12-17

**Authors:** Shengzheng Kuai, Xinyu Guan, Weiqiang Liu, Run Ji, Jianyi Xiong, Daping Wang, Wenyu Zhou

**Affiliations:** ^1^Department of Orthopedics, Affiliated Hospital of Shenzhen University, Shenzhen, Guangdong, China; ^2^Department of Orthopedics, Shenzhen Second People's Hospital, Shenzhen, Guangdong, China; ^3^Shenzhen University School of Medicine, Shenzhen University, Shenzhen, Guangdong, China; ^4^Department of Orthopedics, First Affiliated Hospital Sun Yat-sen University, GuangZhou, Guangdong, China; ^5^Department of Mechanical Engineering, Tsinghua University, Beijing, China; ^6^Key Laboratory of Human Motion Analysis and Rehabilitation Technology of the Ministry of Civil Affairs, National Research Center for Rehabilitation Technical Aids, Beijing, China

## Abstract

**Background:**

Low back pain (LBP) continues to be a severe global healthy problem, and a lot of patients would undergo conservative or surgical treatments. However, the improving capacity of spinal load sharing during activities of daily living (ADLs) after interventions is largely unknown. The objective of this study was to quantitatively predict the improvement of spinal musculoskeletal loadings during level walking and stair climbing after two simulated interventions.

**Material and Methods:**

Twenty-six healthy adults and seven lumbar disc herniation patients performed level walking and stair climbing in sequence. The spinal movement was recorded using a motion capture system. The experimental data were applied to drive a musculoskeletal model to calculate all the lumbar joint resultant forces and muscle activities of seventeen main trunk muscle groups. Rehabilitation and reconstruction were selected as the representative of conservative and surgical treatment, respectively. The spinal load sharing after rehabilitation and reconstruction was predicted by replacing the patients' spine rhythm with healthy subjects' spine rhythm and altering the center of rotation at the L5S1 level, respectively.

**Results:**

During both level walking and stair climbing, the joint resultant forces of the lower lumbar intervertebral discs were predicted to reduce after the two simulated inventions. In addition, the maximum muscle activities of the most trunk muscle groups decreased after simulated rehabilitation and conversely increased after simulated reconstruction.

**Conclusion:**

The predictions revealed the different compensatory responses on the spinal load sharing after two simulated interventions, severing as guidance for making preoperative planning and rehabilitation planning.

## 1. Introduction

According to the report in the literature, low back pain (LBP) continues to be one of the most serious global health problems [1] and causes tremendous direct and indirect economic costs [2–4]. One of the explanations for LBP is disc prolapse inducing nerve root compression. In most cases, the herniation could recover naturally [5], but there are still 5% to 10% of patients with disc herniation who would undergo surgery [6].

Spinal reconstruction has emerged as an effective method to restore the mechanical stability and prevent further pathological development. However, the center of rotation (COR) [7–9], which is a measure of spinal motion quality, will be altered by spinal reconstruction. It has been reported that the alternation of lumbar COR could cause considerable changes in muscle forces using a musculoskeletal model [10]. In addition, the finite element analysis has also shown that the facet forces, ligament loads, and disc stresses are strongly correlated with the location of COR [11]. In an *in vitro* study, it was found that the higher position of COR correlated with the lower facet force [12]. However, most relevant studies only focused on the single spinal functional unit (FSU) such as L5S1 [13, 14]. Additionally, the musculoskeletal model, the finite element model, and cadaveric lumbar spine were usually driven by some fixed load conditions or default constant spine rhythm. To the authors' knowledge, none of the previous studies have reported the effect of different COR locations on the load sharing of the five FSUs' joint resultant forces and multiple trunk muscle groups' activities during level walking and stair climbing.

According to Panjabi's theory [15], the stabilizing system of spine included two subsystems: (1) the spinal column and (2) the spinal muscles. Spinal reconstruction could directly change the motion characteristics of the spinal column. The strength or activation pattern of spinal muscles could be improved by effective rehabilitation. It has been found that rehabilitation for LBP patients would lead to greater improvement in the flexion relaxation response of back muscles [16] and change the muscle onsets of the lumbar erector spinae [17]. In addition, muscle activation to improve trunk stability by rehabilitation was highly related with rehabilitation strategies [17–21]. Since the origin and insertion of every muscle fascicle were around the spinal column, the resultant muscle activity was strongly associated with the spine rhythm [22]. The spine rhythm was denoted by each lumbar segmental motion contribution to the total lumbar motion. Both reconstruction and rehabilitation could improve the spinal load sharing. However, it is not clear which treatment is better to improve the spinal load sharing and to what extent the two kinds of treatment could improve the spinal load sharing.

Therefore, the objective of this study was to quantitatively predict the improvement of joint resultant forces of five lumbar intervertebral discs and muscle activities of seventeen main trunk muscle groups during level walking and stair climbing after simulated rehabilitation and simulated reconstruction for LBP caused by lumbar disc herniation (LDH). In this study, the recovery to healthy people's spine rhythm was considered as the ideal result of rehabilitation. Therefore, rehabilitation was simulated by replacing the patients' spine rhythm by healthy people's spine rhythm. The spinal reconstruction was simulated by changing the position of COR in the musculoskeletal model. Thus, we have the following two hypotheses:

1. The spine rhythm in healthy people would be better for spinal load sharing than that in LDH patients
2. The redistribution of lumbar joint resultant forces and main muscle groups' activities after simulated reconstruction was different from that after simulated rehabilitation

## 2. Methods

### 2.1. Subject

Twenty-six healthy male adults (age: mean 23.6 years (SD 1.92 years), height: mean 169.9 cm (SD 5.9 cm), weight: 63.5 kg (SD 8.4 kg)) and seven male LDH patients (age: mean 28.7 years (SD 4.5 years), height: mean 170.1 cm (SD 3.4 cm), weight: mean 67.4 kg (SD 5.3 kg)) were recruited for this study. The healthy controls were included if they were reported no visible motor dysfunction, no back pain, no surgery in recent one year, and no intense exercise 24 hours before trial. The patient groups were included if: (1) they had the ability to conduct basic activities of daily living such as level walking; (2) they were suffering lumbar disc herniation; (3) the herniation occurred in the lower lumbar region; and (4) the symptom had reached the criteria for surgery. In this study, the disc herniation was found to happen at the L4L5 level in three-seventh cases, at the L5S1 level in another three-seventh cases, and at both L4L5 and L5S1 levels in one-seventh cases. This study was approved by the department of orthopedics of Shenzhen Second People's Hospital in China. All the participants were given informed consent before trial.

### 2.2. Experimental Protocol

In this study, the spinal and pelvic movements were captured by placing the optical markers on the bony landmarks. The bony landmarks included the spinous processes of the third and seventh thoracic vertebra (T3 and T7) and of the first, third, and fifth lumbar vertebra (L1, L3, and L5) and left and right posterior superior iliac spine (LPSIS and RPSIS) and the iliac crest (IC) [23, 24]. Before trials, one surgeon helped to find these landmarks and place the optical markers. Then, individuals were instructed to walk at self-selected, roughly constant speed with a moderate range of arm swing. Subsequently, participants were guided to stand on the ground in front of the staircase and then climb the staircase at a self-selected pace and place only one foot on each staircase. Before data collection, the participants had to practice the two activities until they felt they could perform them naturally.

Before trial, the participant maintained a neutral upright standing position for at least five seconds to collect the baseline data. Then, the participants performed level walking and stair climbing in sequence. Each activity was repeated three times. During the two activities, the markers were captured by Optotrak Certus motion analysis system (Northern Digital Inc., Ontario, Canada) at the sample rate of 100 Hz.

### 2.3. Musculoskeletal Model

In the AnyBody Managed model repository (AMMR, version 1.6) of an AnyBody Modeling system, a generic FacetJointModel was selected since it could predict the muscle forces and intradiscal forces in a redundant system. The details of the model were described and validated in the literature [25–27]. In brief, the model consisted of one pelvic segment, five lumbar vertebrae, and one lumped thoracic segment. The connection between the adjacent segment was a spherical joint which was a simplified model of the intervertebral disc. The location of each joint referred to the work by Pearcy and Bogduk [28]. In this model, there were more than one hundred muscle fascicles around pelvis and spine. These muscles were divided into several main muscle groups based on its function, namely, rectus abdominis (RA), left and right erector spinae (ES), left and right lumbar multifidus (LM), left and right thoracic multifidus (TM), left and right oblique externus (OE), left and right oblique internus (OI), left and right psoas major (PM), left and right quadratus lumborum (QL), and left and right semispinalis (SS). All the muscles fascicles were solved as a force component using the minimum-maximum optimization algorithm and could only exert tensile force [25, 26, 29].

### 2.4. The Spine Rhythm and Simulation

**Figure 1:**
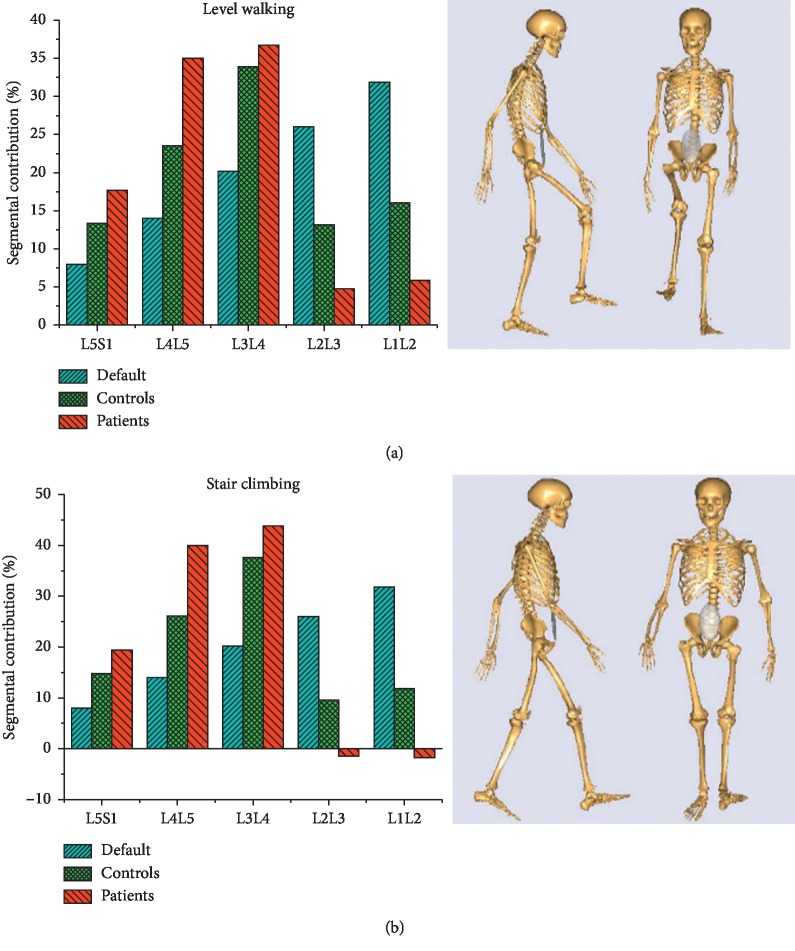
Three types of mean spine rhythm and schematic during (a) level walking and (b) stair climbing.

In the spine model, the motion of every segment was driven by the spine rhythm which represented the contribution of every segment to the total spinal motion. In the AnyBody model system, the default spine rhythm was constant without consideration of the individual difference. In this study, every subject's spine model was driven by the individual spine rhythm which was determined by captured marker coordinates [23]. The features of default, control group, and patient group's spine rhythms during level walking and stair climbing were represented by the average across the gait cycle and are shown in Figure 1.

### 2.5. The Selection of the Optimized COR and Secondary Simulation

**Figure 2:**
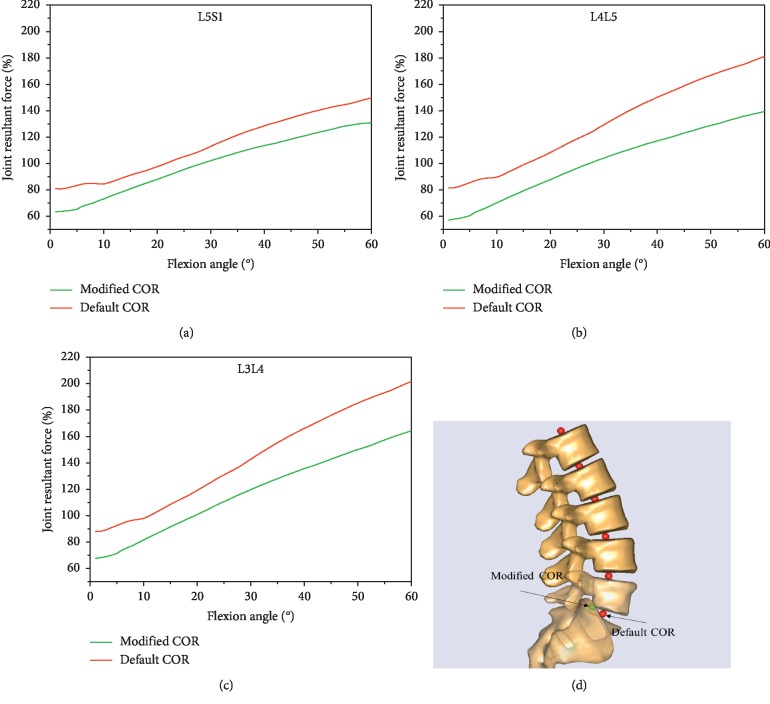
The position of the modified center of rotation and predicted the joint resultant forces at L3L4, L4L5, and L5S1 for default and modified center of rotation. The green line indicates the modified center of rotation. The red line indicates the default center of rotation.

In this study, the position of COR was set offset from default COR for L5S1 (Figure 2). The offset was from 10 mm anterior to 10 mm posterior with an interval of 1 mm and 10 mm inferior to 10 mm superior with an interval of 1 mm. Then, the musculoskeletal model was driven to perform trunk flexion using the default lumbar spine rhythm under each offset COR. During simulation, the intradiscal forces were recorded. Finally, one position of the COR at L5S1 was selected because the joint resultant forces at L3L4, L4L5, and L5S1 levels were significantly decreased (Figure 2). Afterward, the musculoskeletal model was modified by resetting the default COR with new COR and then driven by every patient's spine rhythm during level walking and stair climbing.

### 2.6. Data Analysis

For the two ADLs, the gait cycle was defined as the time interval between subsequent heel strikes of the same leg. Then, the data of muscle activities and intradiscal forces were intercepted by the time window of the analyzed cycle. The intercepted data were time-normalized to 0–100% with 101 points. Moreover, the intradiscal forces were normalized to the body weight of every subject. The improvement at each time point was determined by the value of the variable before intervention subtracting from the value of the variable after the intervention. Data analysis was performed using custom-made programs implemented in MATLAB (the MathWorks, Inc.).

## 3. Results

### 3.1. The Effect of Two Simulated Interventions on the Joint Resultant Forces during Level Walking and Stair Climbing

**Figure 3:**
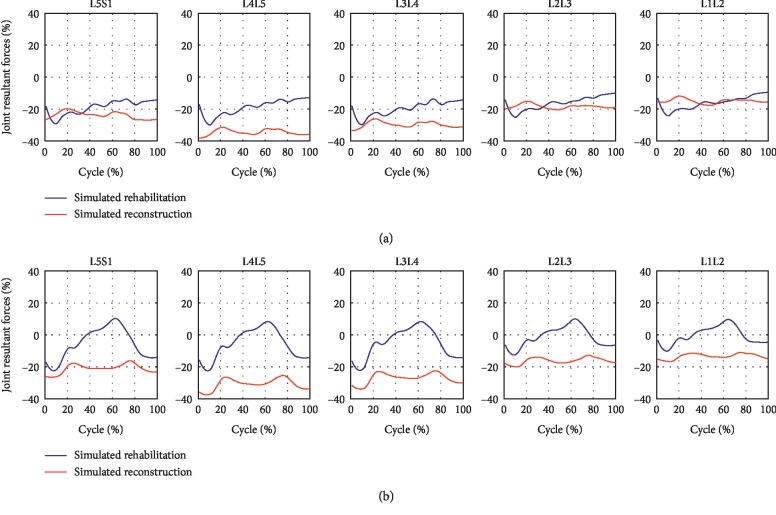
The improvement of joint resultant forces during (a) level walking and (b) stair climbing after the two interventions.

Figure 3 illustrates the effect of two interventions on joint resultant forces during level walking and stair climbing. During level walking, the two interventions both decreased the joint resultant forces acting on all the five lumbar intervertebral discs. However, there were more decreases after simulated reconstruction than simulated rehabilitation. During stair climbing, all the five lumbar motion segment units showed larger reductions in joint resultant forces throughout the gait cycle after simulated reconstruction, while the improvement after simulated rehabilitation varied across the gait cycle. However, the joint resultant forces acting at the L5S1, L4L5, and L3L4 levels were decreased in average after simulated rehabilitation.

### 3.2. The Effect of Two Simulated Interventions on the Muscle Activities during Level Walking and Stair Climbing

**Figure 4:**
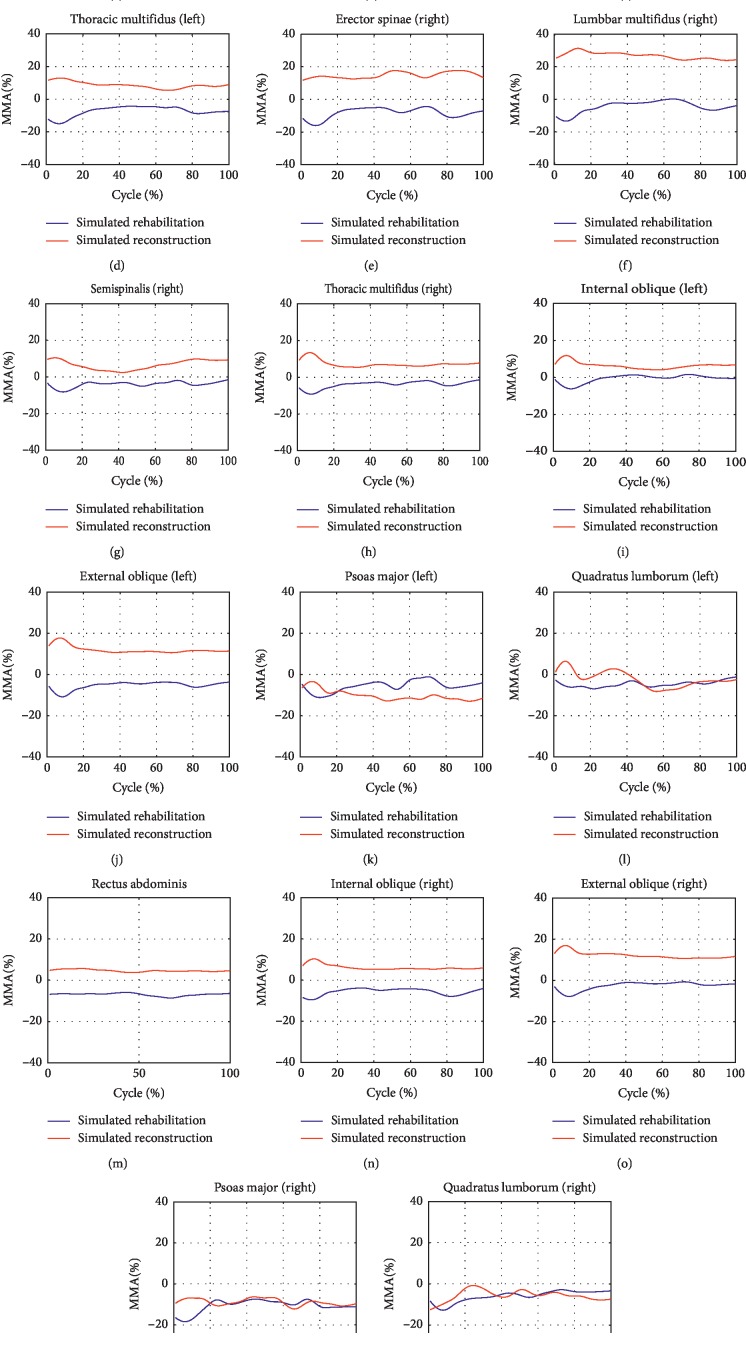
The improvement of maximum muscle activities of the seventeen main trunk muscle groups during level walking after the two interventions.

**Figure 5:**
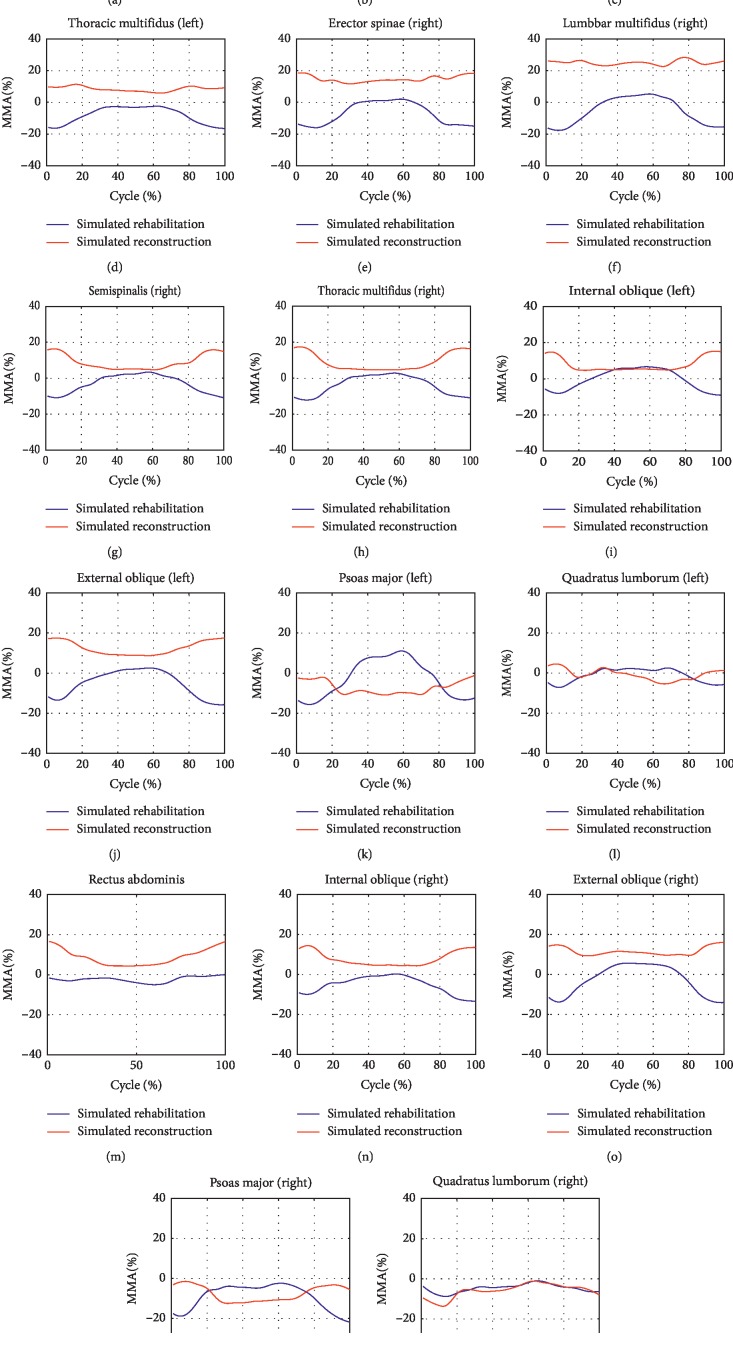
The improvement of maximum muscle activities of the seventeen main trunk muscle groups during stair climbing after the two interventions.

The maximum muscle activities (MMAs) of all the seventeen muscle groups were found to possess reductions during level walking after simulated rehabilitation (Figure 4). In contrast, larger MMAs after simulated reconstruction were found in RA and two sides of ES, LM, SS, TM, IO, and EO. During stair climbing, the MMAs of all the back muscle groups and five of the nine front muscle groups were improved after simulated rehabilitation (Figure 5). In contrary to simulated rehabilitation, there were increases in the MMAs of all the back muscle groups after simulated reconstruction. In addition, seven of the nine front muscle groups showed more MMAs after simulated reconstruction.

## 4. Discussion

The goal of this study was to predict the effect of two simulated interventions for LDH on the intradiscal forces acting on the five lumbar intervertebral discs and maximum muscle activities of seventeen main muscle groups in the spinal region during two common ADLs.

The findings showed that there were reductions in the joint resultant forces at L5S1, L4L5, and L3L4 levels and the MMAs of the majority of the seventeen muscle groups during the two ADLs after simulated rehabilitation, supporting the first hypothesis. More decreases were found in joint resultant forces after simulated reconstruction than simulated rehabilitation during the two ADLs. In addition, the majority of the seventeen muscle groups demonstrated smaller MMAs after simulated rehabilitation but larger MMAs after simulated reconstruction. These findings supported the second hypothesis.

In the first hypothesis, the reductions of joint resultant forces were expected since decompression was one of the main therapeutic purposes for LDH. Apart from decompression, the muscle activities were also decreased after simulated rehabilitation, which was also expected since LBP could induce increases in lumbar muscle activities during functional tasks [30, 31]. The improvement in joint resultant forces and trunk muscle activities may be explained within the context of proper spine rhythm. To perform specific ADL, the central nervous system (CNS) would allocate motion for every FSU. The spinal column and musculature, which were two primary stabilizing systems in Panjabi's model of spinal stability, would respond for the motion allocation. In Arjmand et al.'s studies [22, 32], subjective alteration of spine rhythm in healthy people induced the changes in the spinal loads and muscle activities, which was in consistent with Panjabi's spinal stabilizing theory. In patients with LDH, the CNS adopted different spine rhythm strategies (Figure 1) due to subjective fear or habitual protective behavior. Likely, the spinal column and musculature exhibited adaptive response for this spine rhythm. However, this adaptation imposed extra burdens on lumbar discs and trunk muscles because the healthy spine rhythm might be an optimal strategy for diminishing compression force [33]. Moreover, this study found that healthy spine rhythm could also lowered the burden of musculature. So, the final purpose of rehabilitation might be the recovery of the healthy spine rhythm.

In the second hypothesis, the joint resultant forces were decreased after the alteration of COR. Additionally, the effect of decompression was better after simulated reconstruction than simulated rehabilitation. The simulated rehabilitation changed the motion distribution on FSU, but the structure and motion quality of every FSU were not altered. However, the structure of FSU, which was deemed as the base of the spine, was restored after simulated reconstruction. The restoration of the spinal base might contribute to better decompression. Noteworthy was that the obvious decompression in the lower lumbar region occurred under both default spine rhythm and patient' spine rhythm even though the two rhythms are quite different (Figure 1). It could be concluded that the alternation of COR played a greater role in decompression than the alteration of spine rhythm. Different with that after simulated rehabilitation, the decreases in joint resultant forces were accompanied by the increases in MMAs of the most main trunk muscle groups, especially in back muscle groups. Han et al. [10] have also reported that the muscle forces and activation patterns could be strongly affected by the location of COR, inducing considerable higher muscle forces. In this study, the COR moved posteriorly. The lever arms of the back muscle fascicles became shorter. So, to stabilize the spinal system, it is an adaption to increase muscle forces drastically.

In closing, understanding the load sharing in the spinal region and grading the load conditions would be beneficial for the selection of treatment in clinical examination. Our findings show that both interventions could reduce the joint resultant forces. However, in consideration of huge tissue injuries and possible extra burden on muscles caused by COR offset after reconstruction, rehabilitation should be a prior intervention. Reconstruction would be advised when the larger decompression was essential. In the future study, we will assess every patient's spinal load sharing pattern and find the correlation between spinal load sharing pattern and the therapy effect, which would guide the clinical treatment in specific.

## 5. Limitations

In this study, there were several limitations. Firstly, reduction in joint resultant forces was selected as the inclusive criteria for optimal COR, which might be a little arbitrary. The optimization of COR should take intradiscal forces, facet forces, muscle forces, and ligament forces into consideration. However, it was difficult to allocate weight for every variable. So, the selection for optimal COR was just a simplified method. Secondly, the prediction for reconstruction omitted the tissue injuries caused by surgery, which might affect the amplitude of some muscle forces. Thirdly, the interaction between spine rhythm and COR was not taken into consideration.

## 6. Conclusions

This study shows that both simulated rehabilitation and simulated reconstruction would affect the load sharing in the spine stabilizing system. Spinal loading decrease for lumbar intervertebral discs in the pathological region was predicted after both interventions. However, simulated rehabilitation reduced the muscle activities, while simulated reconstruction increased muscle activities. Considering the rapid decompression and better effects after simulated reconstruction and improvement of muscle activities after simulated rehabilitation, the combination of reconstruction and rehabilitation might be a better treatment choice for severe LDH patients. Besides, the prediction of loading characteristics during ADLs after two interventions might provide a crucial insight into the preoperative planning and rehabilitation planning.

## Data Availability

The data used to support the findings of this study were supplied by Dr. Wenyu Zhou under license and so cannot be made freely available. Requests for access to these data should be made to the corresponding author Dr. Wenyu Zhou.
